# The Separative Performance of Modules with Polymeric Membranes for a Hybrid Adsorptive/Membrane Process of CO_2_ Capture from Flue Gas

**DOI:** 10.3390/membranes10110309

**Published:** 2020-10-28

**Authors:** Aleksandra Janusz-Cygan, Jolanta Jaschik, Artur Wojdyła, Marek Tańczyk

**Affiliations:** Institute of Chemical Engineering, Polish Academy of Sciences, ul. Bałtycka 5, 44-100 Gliwice, Poland; jjaschik@iich.gliwice.pl (J.J.); artur.wojdyla@iich.gliwice.pl (A.W.); mtanczyk@iich.gliwice.pl (M.T.)

**Keywords:** polysulfone and polyimide membrane, CO_2_ capture, hybrid process, multicomponent membrane separation, mathematical modeling

## Abstract

Commercially available polymeric membrane materials may also show their potential for CO_2_ capture by the association of the membrane process with other separation techniques in a hybrid system. In the current study, PRISM PA1020/Air Products and UBE UMS-A5 modules with membrane formed of modified polysulfone and polyimide, respectively, were assessed as a second stage in the hybrid vacuum swing adsorption (VSA)–membrane process developed in our laboratory. For this purpose, the module permeances of CO_2_, N_2_, and O_2_ at different temperatures were determined, and the separation of CO_2_/N_2_ and CO_2_/N_2_/O_2_ mixtures was investigated in an experimental setup. An appropriate mathematical model was also developed and validated based on experimental data. It was found that both modules can provide CO_2_-rich gas of the purity of > 95% with virtually the same recovery (40.7−63.6% for maximum carbon dioxide content in permeate) when fed with pre-enriched effluent from the VSA unit. It was also found that this level of purity and recovery was reached at a low feed to permeate the pressure ratio (2−2.5) in both modules. In addition, both modules reveal stable separation performance, and thus, their applicability in a hybrid system depends on investment outlays and will be the subject of optimization investigations, which will be supported by the model presented and validated in this study.

## 1. Introduction

The problem of reducing greenhouse gas emissions is of profound social and scientific importance. The main culprit in provoking climate change is attributed to carbon dioxide emitted into the atmosphere in various industrial processes and, most notably, by the energy sector. The capture of CO_2_ may be realized using well-established gas separation techniques, including absorption, adsorption, membrane separation, and cryogenic processes [1,2,3,4,5,6,7,8]. It is expected that a system accomplishing the separation of CO_2_ from flue gases generated during the combustion of fossil fuels should provide an enriched stream with CO_2_ concentration exceeding 95 vol.% and CO_2_ recovery above 90% [9]. A number of technologies that fulfill these criteria have been developed [10]. Currently, the most mature and commercially attractive techniques are based on absorption. Most of the pilot plants for the capture of CO_2_ from the flue gas is based on this process. However, other techniques are still being considered, taking into account their flexibility, in terms of feed stream specifications and operating conditions, high selectivity of the carbon dioxide vs. nitrogen and oxygen, more efficient regeneration as is in the case of adsorption, or even the lack of the regeneration step as is in the case of membranes.

In the case of membrane separation, the Technology Readiness Level is 5 (on the 1 to 9 scale); this means that pilot-scale processes are proposed in which around 1 t of CO_2_ per day may be removed [10,11]. However, the possibility to combine these techniques with existing or new power units is rather limited, mainly due to the energy use exceeding that in absorptive methods [2] and the large size of the relevant equipment. As has been pointed out by Merkel et al. [12], to remove 90% of CO_2_ emitted by a 600 MW_e_ power plant, it is necessary to use 1.3−2 Mm^2^ of a membrane with a CO_2_ permeability of 1000 GPU and a CO_2_/N_2_ ideal separation factor of 50. Moreover, such a process consumes 16–24% of the energy produced.

For the last few years, vast effort has been made in order to prepare membrane materials compatible with technical and economic requirements of a viable CO_2_ capture process [12,13,14,15]. However, the way from the development of a new membrane material to its practical application and commercialization is very long. In one of a few cases, the new MTR system with the Polaris^TM^ membrane (Membrane Technology and Research, Inc., Eureka Drive Newark, CA, USA) have successfully passed a long-term test (7000 h) in a laboratory and pilot installation on real combustion gases (1 MW_e_ scale) [3,12,16]. Taking into account other membrane separation processes, more than 90% of current commercial membranes are made of materials used for decades [17] despite the synthesis and evaluation of thousands of new ones. Moreover, a vast majority of practical membrane separations are based on polymeric membranes in which the solution-diffusion mechanism of gas transport prevails.

An interest in polymeric membrane materials available on the market in the context of CO_2_ capture is being maintained by the association of a membrane process with other separation techniques in a hybrid system that may combine, for example, membrane and absorption [18,19,20], membrane and cryogenic separation [21,22,23], or adsorption and membrane [24,25,26]. Our group developed a hybrid technique for CO_2_ capture from flue gas in which the first stage includes a four-column vacuum swing adsorption (VSA) installation, and the second stage is a membrane module available on the market [25,27]. The first stage of this system produces two streams: purified gas and CO_2_-enriched gas. This stage is oriented to assure a high recovery of carbon dioxide at the expense of a limited CO_2_ concentration in the discharged pre-enriched gas. Therefore, in the case of a VSA unit, high vacuum in the bed regeneration steps of the VSA cycle can be avoided [28]. Since most of the ballast (nitrogen and oxygen) is discharged from the system as the purified gas, and the membrane module is fed with the CO_2_-enriched gas, it becomes possible to lower the compression work [29], which provides a driving force to the gas permeation. It has been theoretically shown that, in such a hybrid system, which was fed with a mixture of CO_2_ 13.3 vol.%, N_2_ 80.7 vol.%, and O_2_ 6 vol.%, the energy consumption may be lowered well below 2 MJ_e_ kg_CO2_^−1^ while capturing over 90% of carbon dioxide in a stream of >9% CO_2_ purity [26].

In the hybrid system presented in Warmuzinski et al. [25] and Jaschik et al. [26], the PRISM PA1020/Air Products (Air Products and Chemicals, Allentown, PA, USA) module with a membrane formed of modified polysulfone was used. The module originally designed for the separation of air was successfully used in the hybrid system after some preliminary tests, assuming the CO_2_ concentration at the module inlet of ~70 vol.% [25,26]. However, comprehensive data concerning separation properties of the module in this specific case is required in order to formulate and validate a mathematical model of the process and/or perform the VSA–membrane process optimization. Such data is usually not available in the case of the modules available on the market. Therefore, in this paper, the separation properties of the polysulfone membrane in the context of CO_2_ capture from flue gases were comprehensively analyzed and compared with those of the polyimide one as implemented in the UBE UMS-A5 (Ube Industries, Ltd., Tokyo, Japan) module. The results of extensive experimental studies on the permeation of pure gases and a study of the separation of binary and ternary gas mixtures containing the main components of the dry flue gas stream were presented.

In the theoretical study of the VSA–membrane hybrid system [26], a mathematical model was presented in which the membrane stage was modelled assuming a plug flow on the feed side and a locally unhindered flow on the permeate side. However, a flow pattern in the UBE UMS-A5 module is better described by plug flow on the feed side and countercurrent plug flow on the permeate side. Therefore, the appropriate mathematical model was formulated in order to simulate multicomponent gas permeation in this module. The model and the results of its validation against the experimental data was also presented in this study.

## 2. Experimental Procedure and Mathematical Modeling

### 2.1. Experimental Setup

**Figure 1:**
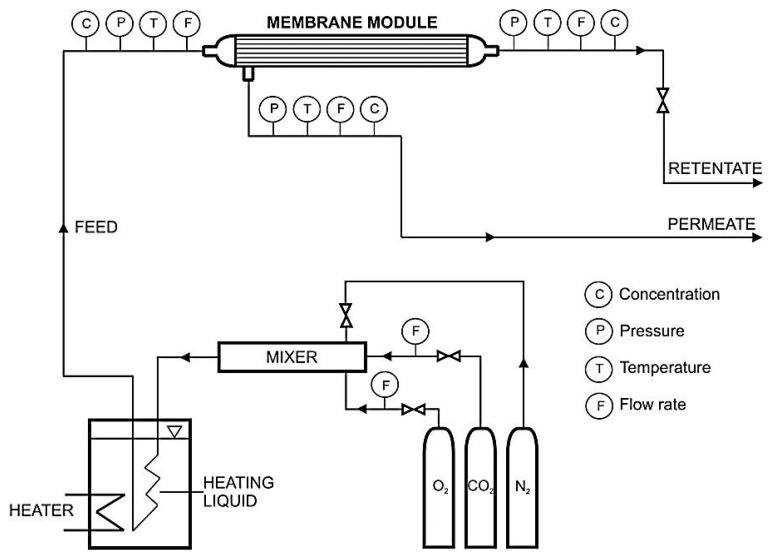
Experimental setup for gas permeation studies.

Gas permeation studies were performed in an experimental setup with an exchangeable membrane module, which is presented schematically in Figure 1. The setup is equipped with the gas preparation section, which consists of gas cylinders, mixer and thermostat. The gas from one or more gas cylinders passes through a mixer, which is a steel column with a diameter of 20 mm and a length of 500 mm. Its temperature was further established in the water thermostat (Julaba F12, accuracy of temperature stabilization: 0.1 °C) before entering the membrane module. The module and the feed gas, permeate, and retentate lines are isolated. Pressure, temperature, feed flow rate, and composition (in the case of gas mixtures) were measured and recorded during experiments. In the latter case, the three-channel Varian (CP-4900, Varian Inc., Palo Alto, CA, USA) microchromatograph was used. At the same time, the concentration of the mixture components was measured in three gas streams with an accuracy of 0.01 vol.%. The pressure was measured with pressure transducers (Cole–Parmer P series, Cole−Parmer Instrument Company, Vernon Hills, IL, USA) with an accuracy of 0.1 psi. For the temperature measurement, the Cole–Parmer Digi-Sense was used, which provided an accuracy of 0.1 °C. Aalborg GFM37 (Aalborg Instruments, New York, NY, USA) flow meters with an accuracy of 0.1 L min^−1^ were used to measure the feed gas, retentate, and permeate flow rates.

### 2.2. Membrane Modules

Two hollow-fiber modules were used: PRISM PA1020, provided by Air Products and UMS-A5, provided by UBE. The first one, designed mainly for air separation, has a membrane formed of modified polysulfone. In the UBE’s module, also designed mainly for air separation, a polyimide membrane was used. The modules were used in the experimental setup as supplied, and parameters used in the experiments were within the pressure and temperature ranges recommended by the manufacturers. No data concerning membrane area or thickness was disclosed by the providers. The technical parameters of the modules are given in the Supplementary material (Table S1).

### 2.3. Gas Permeation Studies

Permeation investigations were performed for the pure main components of flue gas streams (CO_2_, N_2_, and O_2_) and their mixtures. In the case of pure gases, the feed gas pressure was varied in the range of 1.2–7.5 bar (abs) at a set temperature in the range of 288–298 K, and the corresponding permeate flow rate was measured. The absolute permeate pressure was close to the ambient pressure but was recorded for every single experimental point. The permeate flow rate is a basis for the determination of the module gas permeance, which is understood here as a product of the permeance and the membrane area, which is unknown in the investigated cases,

(1) $$AQ\;=\;\frac{F_{P}}{p_{Z}-p_{P}}\;$$

where *A* is the membrane area, m^2^; *Q* is the permeance, kmol h^−1^ bar^−1^ or GPU; *F_P_* is the permeate flow rate, kmol h^−1^; and *p_Z_* and *p_P_* are the feed pressure and permeate pressure, respectively, bar.

The ideal separation factor (α) of nitrogen and oxygen vs. carbon dioxide may be defined in this case as

(2) $$\propto_{i/CO2}=\frac{AQ_{i}}{AQ_{CO2}}\;$$

Although *AQ* is independent of the feed gas flow rate, the permeation of pure gases was measured at three feed gas flow rates of 0.025, 0.05, and 0.075 kmol h^−1^ to check for experimental errors as well as for consistency of the setup and methodology.

The separation of two component mixtures containing 10, 40, and 70 vol.% of carbon dioxide in nitrogen was investigated experimentally for the feed gas flow rate of 0.05 kmol h^−1^ and the temperature of 293–295 K. For carbon dioxide concentration of 70 vol.%, the impact of oxygen for the separation process at the same flow rate and the temperature of 297−298 K was also investigated. Mixtures containing 2 vol.% and 5 vol.% of oxygen were used. The performance of the separation process was evaluated on the basis of CO_2_ concentration in the permeate and the carbon dioxide recovery,

(3) $$Recovery=\frac{y_{CO2}\;F_{P}}{x_{Z,CO2}\;F_{Z}}\;$$

where *y* is the mole fraction on the permeate side; *x_Z_* is the mole fraction on the feed side; and *F_Z_* is the feed gas flow rate, kmol h^−1^.

### 2.4. Mathematical Model

**Figure 2:**
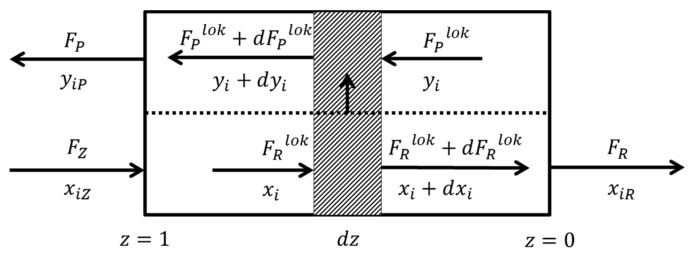
Gas streams in a membrane module for plug flow on the feed side and countercurrent plug flow on the permeate side.

Mathematical model concerning the separation of CO_2_/N_2_ and CO_2_/N_2_/O_2_ mixtures in the membrane module was formulated based on the model presented by Sengupta and Sircar [30,31], which reflects the flow pattern presented in Figure 2. A fairly simple model was selected taking into account that it will serve as a model of a unit process in a complex model concerning the hybrid system [26]. Plug flow was assumed on both membrane sides. It means that the concentration gradient perpendicular to the gas flow direction is not considered. It was also assumed that there are no interactions between the permeating components (therefore, the permeances are the same as for pure components), the pressure drop and the axial dispersion are negligible on both sides of the membrane, the process is isothermal and the concentration polarization is also negligible on both sides of the membrane.

For a three-component gas mixture, the gas composition on the feed side is determined from the following equations:

(4) $$\frac{dx_{1}}{dz}=\frac{2\;K\;\left[x_{1}\;\left(C_{1}-B\right)-\gamma \;\left(C_{1}\;y_{1}-x_{1}\;E\right)\right]}{F_{R}^{*}}\;$$

(5) $$\frac{dx_{2}}{dz}=\frac{2\;K\;\left[x_{2}\;\left(C_{2}-B\right)-\gamma \;\left(C_{2}\;y_{2}-x_{2}\;E\right)\right]}{F_{R}^{*}}\;$$

(6) $$x_{3}=1-x_{1}-x_{2}\;$$

where

(7) $$K=\left(\overset{3}{\underset{i=1}{\sum }}AQ_{i}\right)\;\frac{p_{Z}}{F_{Z}}\;$$

(8) $$C_{i}=\frac{AQ_{i}}{\sum_{i=1}^{3}\left(AQ_{i}\right)}\;$$

(9) $$B=C_{3}+\left(C_{1}-C_{3}\right)\;x_{1}+\left(C_{2}-C_{3}\right)\;x_{2}\;$$

(10) $$E=C_{3}+\left(C_{1}-C_{3}\right)\;y_{1}+\left(C_{2}-C_{3}\right)\;y_{2}\;$$

(11) $$\gamma =\frac{p_{P}}{p_{Z}}\;$$

(12) $$F_{R}^{*}=\frac{F_{R}^{lok}}{F_{Z}}\;$$

where *F_R_* is the retentate flow rate, kmol h^−1^; *z* is the dimensionless module length coordinate; *1*,*2*,*3* is the number of component; and *lok* is the local value.

The dimensionless ratio of the local retentate flow rate and feed gas flow rate, F_R_^*^, defined by Equation (12), is determined from the equation:

(13) $$\frac{dF_{R}^{*}}{dz}=2\;K\;\left(B-\gamma \;E\right)\;$$

Mole fractions of the permeate are derived from

(14) $$\frac{dy_{1}}{dz}=\frac{2\;K\;\left[\left(C_{1}\;x_{1}-B\;y_{1}\right)-\gamma \;y_{1}\;\left(C_{1}-E\right)\right]}{F_{P}^{*}}\;$$

(15) $$\frac{dy_{2}}{dz}=\frac{2\;K\;\left[\left(C_{2}\;x_{2}-B\;y_{2}\right)-\gamma \;y_{2}\;\left(C_{2}-E\right)\right]}{F_{P}^{*}}\;$$

(16) $$y_{3}=1-y_{1}-y_{2}\;$$

where

(17) $$F_{P}^{*}=\frac{F_{P}^{lok}}{F_{Z}}\;$$

The dimensionless ratio of the local permeate flow rate and feed gas flow rate, F_P_^*^, defined by Equation (17), is determined from the equation

(18) $$\frac{dF_{P}^{*}}{dz}=2\;K\;\left(B-\gamma \;E\right)\;$$

Boundary conditions are determined for *z* = 0 (retentate outlet) as

(19) $$y_{1}=\frac{C_{1}\;\left(x_{1R}^{\;}-\gamma \;y_{1}\right)}{C_{3}\;\left(1-\gamma \right)+\left(C_{1}-C_{3}\right)\;\left(x_{1R}^{\;}-\gamma \;y_{1}\right)+\left(C_{2}-C_{3}\right)\;\left(x_{2R}^{\;}-\gamma \;y_{2}\right)}\;$$

(20) $$y_{2}=\frac{C_{2}\;\left(x_{2R}^{\;}-\gamma \;y_{2}\right)}{C_{3}\;\left(1-\gamma \right)+\left(C_{1}-C_{3}\right)\;\left(x_{1R}^{\;}-\gamma \;y_{1}\right)+\left(C_{2}-C_{3}\right)\;\left(x_{2R}^{\;}-\gamma \;y_{2}\right)}\;$$

(21) $$F_{P}^{*}=0\;$$

and for *z* = 1 (feed gas inlet) as

(22) $$x_{1}=x_{1Z}^{\;}\;$$

(23) $$x_{2}=x_{2Z}^{\;}\;$$

(24) $$F_{R}^{*}=1\;$$

For the purpose of this study, this set of first-order ordinary differential equations with accompanying algebraic equations and boundary conditions was implemented and solved in Mathcad. The Runge–Kutta method of the fourth order with a given integration step *dz* was used and the integration started from the retentate outlet and ended at the feed gas inlet.

## 3. Results and Discussion

### 3.1. Module Permeance and Ideal Selectivity

**Figure 3:**
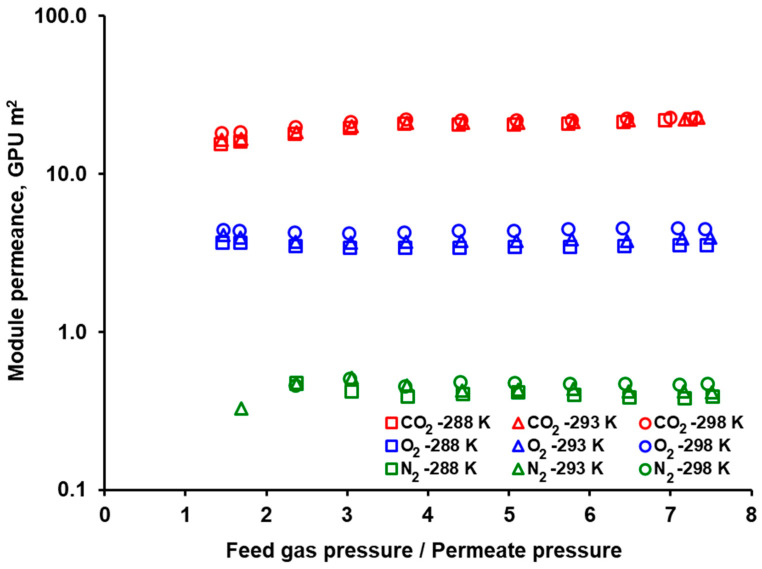
Module permeance of CO_2_, N_2_, and O_2_ in UBE UMS-A5 against the feed to permeate pressure ratio for feed gas flow rate of 0.05 kmol h^−1^.

The module permeances (AQ_i_) of carbon dioxide, nitrogen and oxygen determined from single gas experiments in both investigated modules are presented in Table 1 and Table 2. In the case of UBE UMS-A5, the module permeances are also graphically shown in Figure 3 for a feed gas flow rate of 0.05 kmol h^−1^. As can be seen in that figure, the permeance of nitrogen and oxygen is independent of pressure under the experimental conditions. An increase in the module permeance in the case of nitrogen for the lowest feed to permeate pressure ratio is most probably associated with the fact that the permeate flow rate was very low and could not be measured with sufficient accuracy. In the case of CO_2_, a continuous rise in the module permeance is observed with the increase in the feed pressure. Taking into account that we deal with glassy polymers, this may be caused by the net effect of adsorption/diffusion of CO_2_ in the fractional free volume (FFV). On the one hand, the solubility decreases nonlinearly with pressure, while strong non-linear adsorption occurs in FFV. This is probably the case with CO_2_. On the other hand, the diffusion in FFV is faster than in a solid polymer matrix, and additionally the diffusivity of CO_2_ may increase with pressure. Probably the latter effect prevails in the situation observed in Figure 3. In order to make a convenient comparison, the values of module permeance presented in Table 1 and Table 2 are the averages from the experimental data concerning the three feed gas flow rates of 0.025, 0.05, and 0.075 kmol h^−1^. For better visibility, the error bars are not presented in Figure 3, and the mean standard deviation is given in Table 1 and Table 2.

The permeances of CO_2_, N_2_, and O_2_ measured in our laboratory are in conformity with the data given by Ettouney and Majeed [32] for a similar Air Products PRISM module with a membrane area of 2.24 m^2^. At 297 K, they reported the average CO_2_, N_2_, and O_2_ permeances of 139.99, 3.07, and 20.0 GPU, respectively. Assuming the same membrane area, the permeance determined in this study would be of the same order equal to 161.33, 4.39, and 30.37 GPU at 298 K (cf. Table 2). In the case of a UBE UMS-A5 module with polyimide membrane, Kase et al. [33] reported an ideal CO_2_/N_2_ selectivity of 41 at 298 K, which is comparable to the value measured in this study (45.5). They also showed a CO_2_ permeance of 1300 GPU, which suggests that the module used in the current study has a rather small membrane are of ~167 cm^2^. The circumstance that the difference between the module permeance is a matter of the membrane area rather than the permeability is confirmed by various sources. In the case of polysulfone CO_2_, permeability of 5.6 is reported by references [34,35,36] at 303 K, which is accompanied by CO_2_/N_2_ and CO_2_/O_2_ ideal selectivity of 22 and 4, respectively. In turn, for polyimide and its modifications, carbon dioxide permeability ranging from 0.5 to 61.9, and CO_2_/N_2_ and CO_2_/O_2_ ideal selectivity of 18.4−41 and 2.8−5.6, respectively, was reported at various temperatures in several sources [33,34,35,36,37].

**Figure 4:**
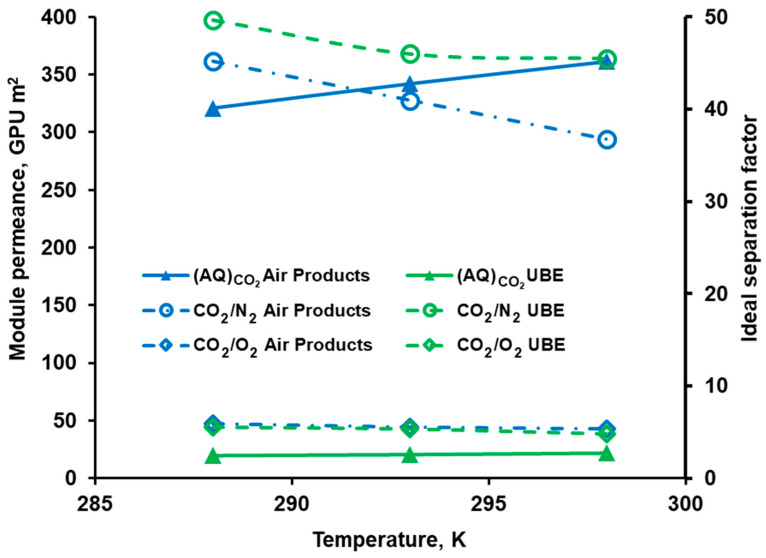
Module permeance of CO_2_ and ideal selectivity of CO_2_/N_2_ and CO_2_/O_2_ in UBE UMS-A5 and Air Products PRISM PA1020 module against temperature.

The module permeance of CO_2_ along with the ideal selectivity is shown as a function of temperature in Figure 4. As can be seen in this figure, the former parameter increases linearly with an increase in temperature. The permeance raise is slightly more visible for Air Products PRISM PA1020 (~11.1% between 288 K and 298 K) than for UBE UMS-A5 (~9.7% between 288 K and 298 K). The latter module reveals a slightly higher CO_2_/N_2_ ideal selectivity of 45.2–50.3 compared to 36.9–45.2 for Air Products PRISM PA1020. The ideal selectivity of CO_2_/O_2_ (~5–6) is comparable for both modules.

The ideal selectivity of CO_2_/N_2_ and CO_2_/O_2_ decreases with increasing temperature in both modules. This is due to the fact that the activation energy of permeation is lower for CO_2_ by comparison to nitrogen and oxygen. As a consequence, less permeable gases show a greater increase in permeability with increasing temperature. This activation energy of permeation was determined from experimental data using the following equation:

(25) $$AQ=AQ_{0}e^{\frac{-E_{a}}{RT}}\;$$

where *Q*_0_ is the pre-exponential factor, kmol h^−1^ bar^−1^ or GPU; *Ea* is the activation energy of permeation, kJ mol^−1^*; R* is the the universal gas constant (8.314 J mol^−1^ K^−1^); and *T* is the temperature, K. In the case of Air Products PRISM, it is equal to 8.5, 16.6, and 23.3 kJ mol^−1^ for CO_2_, O_2_, and N_2_, respectively. In the same order, the activation energy of permeation is equal to 7.4, 16.9, and 13.7 kJ mol^−1^ in UBE UMS-A5.

### 3.2. Separation of CO_2_/N_2_ and Model Validation

**Figure 5:**
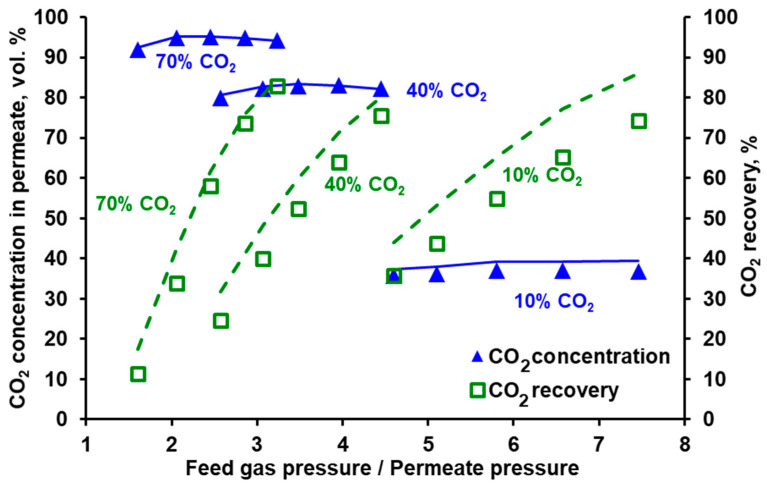
Experimental (points) and theoretical (lines) CO_2_ purity and recovery in Air Products PRISM PA1020 module against feed to permeate pressure ratio for various carbon dioxide content in the feed CO_2_/N_2_ mixture. Feed gas flow rate: 0.05 kmol h^−1^, temperature: 293 K. Standard deviation; purity = ± 0.4, recovery = ± 0.1.

**Figure 6:**
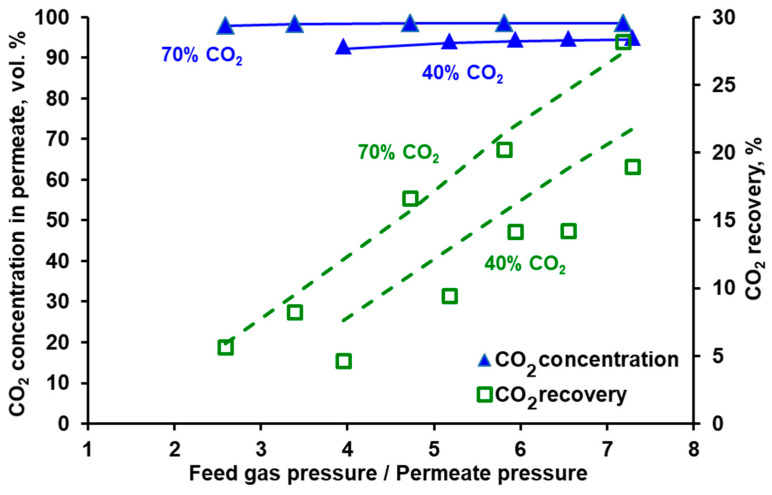
Experimental (points) and theoretical (lines) CO_2_ purity and recovery in UBE UMS-A5 module against feed to permeate pressure ratio for various carbon dioxide content in the feed CO_2_/N_2_ mixture. Feed gas flow rate: 0.05 kmol h^−1^, temperature: 295 K. Standard deviation; purity = ± 0.4, recovery = ± 0.1.

The separation of the carbon dioxide and nitrogen mixture was investigated in both modules for different feed gas flow rates and CO_2_ concentrations at the module inlet. The feed gas flow rate (0.025−0.075 kmol h^−1^) was chosen based on the expected gas flow rate at the inlet to the second stage of the experimental VSA–membrane hybrid system [25]. The experimentally measured CO_2_ content in the permeate and CO_2_ recovery for the three carbon dioxide concentrations in the feed gas are presented as a function of the feed gas and permeate pressures ratio in Figure 5 for Air Products PRISM PA1020 and in Figure 6 for UBE UMS-A5. As can be seen in Figure 5, the CO_2_ concentration in permeate passes through the maximum, while the feed to permeate pressure ratio increases. Moreover, less compression work is necessary when increasing the CO_2_ content at the module inlet. The line of 10 vol.% of CO_2_ at the module inlet is rather flat over a wide range of the feed to permeate pressure ratios, and the CO_2_ purity is at the level of 36–37%. In the context of CO_2_ recovery from flue gases in commercial polymeric membranes, the results presented in Figure 5 are in line with the conclusions reported by other authors. It means that it is not possible to obtain simultaneously both CO_2_ purity and recovery exceeding 80% using a single membrane stage, with CO_2_ concentration at the module inlet below 20 vol.%. And that the compression work can be lowered by increasing this concentration (cf. for example [12,29]).

As can be seen in Figure 5 and Figure 6 it is possible to reach at least 95% CO_2_ purity in both modules when fed with a mixture containing 70 vol.% of carbon dioxide. This points out the necessary composition of the effluent from the vacuum swing adsorption stage in the hybrid system presented in [25,26], if one membrane stage is to be used after a VSA unit.

**Figure 7:**
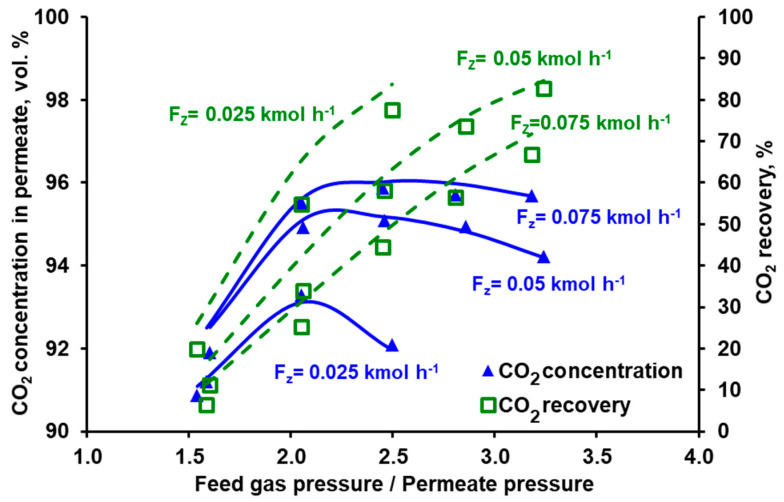
Experimental (points) and theoretical (lines) CO_2_ purity and recovery in Air Products PRISM PA1020 module against feed to permeate pressure ratio for various feed gas flow rates. Inlet CO_2_ concentration: 70 vol.%, temperature: 293 K. Standard deviation; purity = ± 0.4, recovery = ± 0.1.

**Figure 8:**
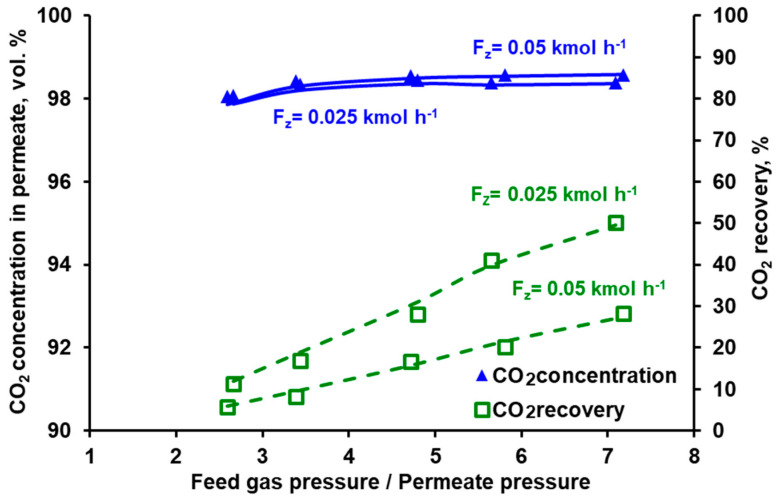
Experimental (points) and theoretical (lines) CO_2_ purity and recovery in UBE UMS-A5 module against feed to permeate pressure ratio for various feed gas flow rates. Inlet CO_2_ concentration: 70 vol.%, temperature: 295 K. Standard deviation; purity = ± 0.4, recovery = ± 0.1.

In the membrane technology, the relation between the product purity and recovery may be tuned by varying the feed gas flow rate, provided that it is within the limit specific for a given membrane module. If the feed gas flow rate is too high, the pressure drop becomes significant, which negatively impacts the driving force of the permeation. If this flow rate is too low, the separation deteriorates significantly, since the impact of axial dispersion starts to be visible and dead spaces can be formed in the module. From this point of view both modules present some flexibility in terms of the inlet feed gas flow rate. In the case of Air Products PRISM PA1020 (cf. Figure 7), an increase in the flow rate at the module inlet leads to a further increase in CO_2_ purity (to ~96%) at the expense of some recovery drop (from 58% to 44.5%). In turn, the recovery in UBE UMS-A5 (cf. Figure 8) can be increased from ~20% to 41% by decreasing the feed gas flow rate from 0.05 kmol h^−1^ to 0025 kmol h^−1^ and at the same time maintaining the CO_2_ concentration in the permeate above 98 vol.%. At the same feed gas flow rate, the CO_2_ recovery reported for Air Products PRISM PA1020 is higher than that for UBE UMS-A5. For example, at 0.05 kmol h^−1^ the recovery is 58.1% for a maximum CO_2_ purity of 95% at a pressure ratio of 2.5 (PRISM PA1020) and 20.2% for a maximum CO_2_ purity of 98.6% at a pressure ratio of 5.8 (UMS-A5). However, this seems to be due to the fact that the area of the PRISM PA1020 module better fits the feed gas flow rates used in the experiments, which in this case correspond to the flow rate of the pre-enriched gas passing from the VSA to the membrane stage in the hybrid installation presented in [25]. The area of UBE UMS-A5 is probably too small in this case, which is also confirmed by the small difference between the CO_2_ concentration in the permeate for different CO_2_ concentrations (cf. Figure 6) and for different feed gas flow rates at the module inlet (cf. Figure 8). Error bars are not presented in Figure 5, Figure 6, Figure 7 and Figure 8 since the mean standard deviation is small (± 0.04 in the case of CO_2_ concentration and ±0.1 in the case of recovery) and would be invisible.

The conducted experiments provided the data necessary to perform a validation of the mathematical model presented in Section 2.4. The results of the numerical simulations shown by the lines in Figure 5, Figure 6, Figure 7 and Figure 8 fit well with the experimental data over the entire range of feed to permeate pressure ratio, carbon dioxide content in the feed gas and gas flow rate at the module inlet. A more visible discrepancy between the model and the experiments can be seen in the recovery for Air Products PRISM PA1020 at the lowest values of CO_2_ concentration and flow rate at the module inlet, as well as for the lowest pressure. In general, the average relative error between the experimental data and the simulation results lies in the range of 2.1–4.0% for CO_2_ concentration and 7.5–28.7% for recovery. The discrepancy observed for the lowest pressure is most probably associated with the low permeate flow rate. On the one hand, the flow rate was measured with less accuracy, and on the other hand, the permeate flow in this case is locally unhindered rather than countercurrent, as assumed in the current model.

### 3.3. Impact of Oxygen on Separation Performance

Binary mixtures of CO_2_/N_2_ are most often considered while assessing the basic separation performance of membrane processes for CO_2_ capture from flue gas, omitting the other main components of flue gas, i.e., water vapor and oxygen. Water vapor was not considered in this study since the dried flue gas is introduced to the VSA–membrane hybrid system [25,26]. This is due to the very high affinity of the zeolite sieve 13X (adsorbent used in the first, vacuum swing adsorption stage) toward water vapor. Therefore, it can be assumed that the second membrane stage is also fed with a dry gas mixture.

The effect of oxygen on CO_2_ membrane capture was presented in our previous study [38]. We have extended discussion presented there in order to have a detailed comparison of both modules and to perform the validation of the model. Oxygen is the least adsorbing component in the VSA stage with ZMS 13X and is mostly removed from the hybrid system with the purified gas. Therefore, in such a situation, the oxygen concentration at the inlet to the membrane stage does not exceed its concentration at the inlet to the hybrid system (e.g., 6 vol.% reported in [26]). However, taking into account that the ideal selectivity of CO_2_/O_2_ is much lower than that of CO_2_/N_2_, even this reduced amount of oxygen can have a significant impact on the performance of the membrane stage.

**Figure 9:**
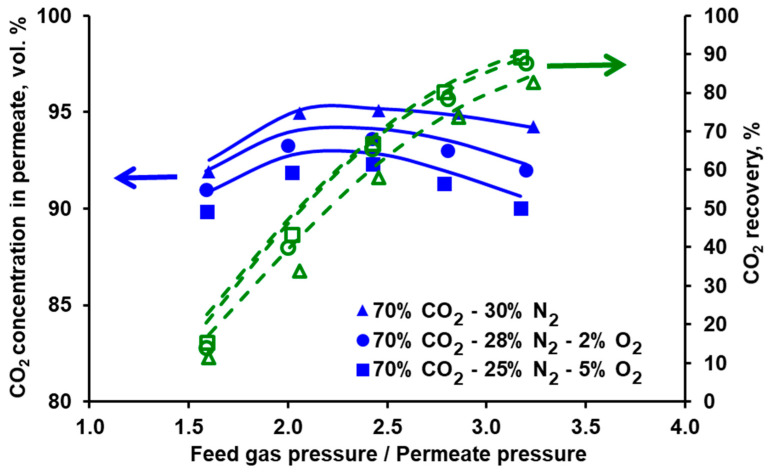
Experimental (points) and theoretical (lines) CO_2_ purity and recovery in Air Products PRISM PA1020 module against feed to permeate pressure ratio for various inlet oxygen concentration. Inlet CO_2_ concentration: 70 vol.%, temperature: 297 K, feed gas flow rate: 0.05 kmol h^−1^.

**Figure 10:**
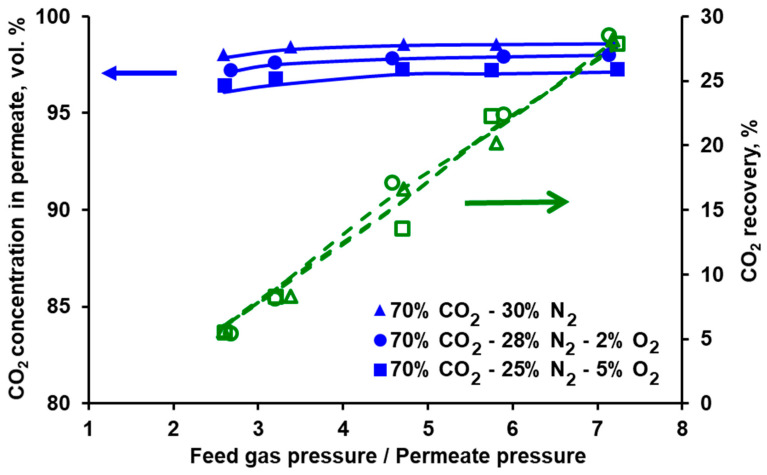
Experimental (points) and theoretical (lines) CO_2_ purity and recovery in UBE UMS-A5 module against feed to permeate pressure ratio for various inlet oxygen concentration. Inlet CO_2_ concentration: 70 vol.%, temperature: 298 K, feed gas flow rate: 0.05 kmol h^−1^.

This can be clearly seen in Figure 9 and Figure 10, where the purity and recovery of CO_2_ in both modules are presented as a function of the feed to permeate pressure ratio for oxygen concentration of 0, 2, and 5 vol.% at the module inlet. The presence of oxygen visibly reduces the carbon dioxide concentration in the permeate with some increase in CO_2_ recovery. The drop in CO_2_ purity is more pronounced for Air Products PRISM PA1020 (from 95.1% to ~90% at a pressure ratio of 2.5 bar) compared to UBE UMS-A5 (from 98.6% to ~97.3% at a pressure ratio of 5.8 bar).

As can be seen in Figure 9 and Figure 10, the results of numerical simulations shown by the lines fit well with the experimental data in the whole range of the process parameters also in the case of the three-component mixture of CO_2_/N_2_/O_2_. The average relative error between the experimental data and the simulation results lies in the range of 0.05–1.1% for CO_2_ concentration and 0.4–13.3% for recovery.

**Figure 11:**
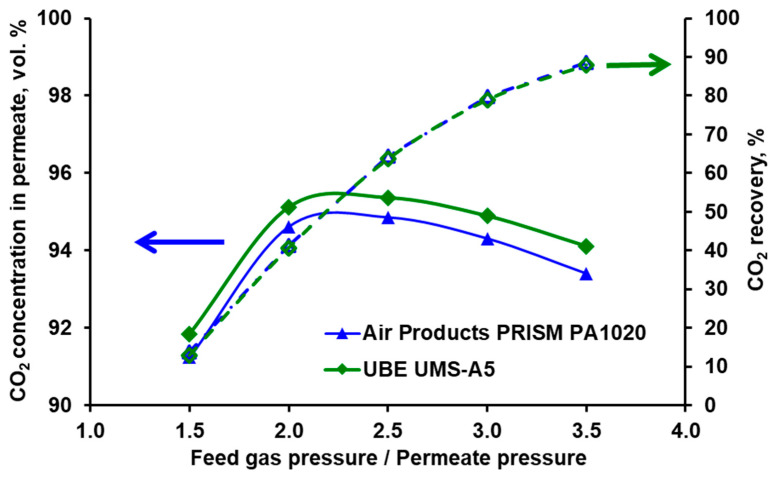
Theoretical CO_2_ purity and recovery in Air Products PRISM PA1020 and UBE UMS-A5 modules against feed to permeate pressure ratio for the feed gas flow rate of 0.05 kmol h^−1^ and 0.0031 kmol h^−1^, respectively. Feed gas: CO_2_ (70 vol.%), N_2_ (28 vol.%), O_2_ (2 vol.%).

The lower sensitivity of the purity (or its practical lack in the case of recovery) to the oxygen content observed in UBE UMS-A5 may be related to the fact that the module is overloaded, as pointed out earlier. In order to make a fair comparison of the both modules, numerical simulations were carried out for a three-component mixture of CO_2_ (70 vol.%), N_2_ (28 vol.%), and O_2_ (2 vol.%) assuming that the permeation number, defined as follows:

(26) $$Permeation\;number=\frac{AQ_{CO2}\;p_{Z}}{F_{Z}}\;$$

is the same for both modules at a given feed pressure and hence feed to permeate pressure ratio. This means that if the feed gas flow rate is 0.05 kmol h^−1^ for Air Products PRISM PA1020 module, it should be reduced to 0.0031 kmol h^−1^ for UBE UMS-A5. The purity and recovery of CO_2_ for both modules are shown in Figure 11 in this case. As can be seen in this figure the characteristics of the separative performance are very similar for both modules except that the UBE UMS-A5 indeed shows a higher CO_2_ purity with virtually the same recovery. However, the difference is smaller when the UBE UMS-A5 works in conditions more appropriate to its area. In the case of the large-scale laboratory hybrid system presented in [25], the UBE module should therefore have at least ten times membrane area in order to fit the enriched-gas flow rate from the VSA unit to the membrane stage. Since such a UBE module on this scale was more expensive than Air Products PRISM PA1020, the economic rationale initially indicated the latter module to be used in a VSA–membrane hybrid system.

As can be seen in Figure 11, the recovery is of 40.7−63.6% within the pressure range of 2−2.5 bar, that is for the highest carbon dioxide concentrations in permeate. However, it should be noted, that it does not represent the recovery of the entire hybrid system since the remaining part of CO_2_ is recycled with retentate to the inlet of the hybrid system [25,26].

### 3.4. Stability of Separation Properties

The separation properties of polymeric membranes may change while exposed to some agents or merely over the time. For example, carbon dioxide can increase the flexibility of the polymer chain, thereby lowering the glass transition temperature of the polymer and plasticizing the membrane. Such plasticization usually worsens the membrane selectivity [39,40,41,42]. If this phenomenon takes place, a parabolic dependence of CO_2_ permeability on pressure is observed, as was shown by Adewole et al. [42] for a polysulfone membrane and the minimum of this parabola points out the plasticization pressure. The latter increases with the thickness of the active membrane layer, as was presented, among others, by Scholes et al. [41] for a polysulfone membrane at 308 K. According to their data, the plasticization pressure lies between 5−8.5 bar with an active layer thickness of 0.25–1 μm, which is the range appropriate for glassy membranes applied in contemporary commercial modules [43].

However, the plasticization induced by carbon dioxide was not observed in our laboratory with either Air Products PRISM PA1020 module or UBE UMS-A5 module. As presented in Figure 3, in the case of UBE UMS-A5, the module permeance of the pure carbon dioxide increases slightly rather than decreases with the feed to permeate pressure ratio over the whole range of feed gas pressure and temperature. Therefore, also from this point of view, both modules can fit very well as a second stage in the hybrid VSA/membrane CO_2_ capture process.

Unlike new membrane materials, the aging of commercial ones is predictable and can be taken into account in the investment process. It is assumed that the lifetime of membranes available on the market is 3–5 years [16,17,44], and the permeability may decrease by at least 30% in the first 3–4 years of the membrane life [17]. The membrane modules presented in this study have been used in our laboratory for several years and tested in various conditions during the design and development of the hybrid VSA–membrane system. Their performance was controlled twice within eight years for Air Products PRISM PA1020 and within seven years for UBE UMS-A5. The corresponding results concerning the module permeance of the pure components are presented in Table 3.

As can be seen in this table, the module permeance of carbon dioxide, and consistently CO_2_ permeability, dropped by 8.8% in Air Products PRISM PA1020 within eight years and by 17.5% in UBE UMS-A5 within seven years. The decrease in permeability is similar for oxygen (12% and 14% for PRISM PA1020 and UMS-A5, respectively) and less pronounced for nitrogen (3.8% and 11.6% for PRISM PA1020 and UMS-A5, respectively). In the case of UBE UMS-A5, the drop-in component permeability is accompanied by a decrease in ideal selectivity (from 47.5 to 44.3, from 5.25 to 5, and from 9 to 8.8 for CO_2_/N_2_, CO_2_/O_2_, and O_2_/N_2_, respectively). Similarly, for Air Products PRISM PA1020, a decrease in CO_2_/N_2_ ideal selectivity from 37.8 to 35.9 was also recorded. Thus, it can be concluded that time and various process conditions have a moderate impact on the separation performance of both modules.

## 4. Conclusions

Two commercially available membrane modules with membranes made of modified polysulfone and polyimide have been comprehensively investigated as a second stage in the vacuum swing adsorption (VSA)/membrane hybrid system for CO_2_ capture from flue gas. It was found that Air Products PRISM PA1020 as well as UBE UMS-A5 fed with pre-enriched effluent from the VSA unit can provide a CO_2_-rich gas of >95% purity, with virtually the same recovery (40.7–63.6% for maximum carbon dioxide content in permeate) when flow rate at the module inlet is matched to their area. This level of purity and recovery was reached with a rather low feed to permeate pressure ratio (2–2.5) in both modules, which can have a positive effect on the energy use in the entire hybrid system. Both modules show a stable separation performance since no plasticization was observed and, moreover, time and various process conditions only moderately affect CO_2_, N_2_, and O_2_ permeances. Thus, their applicability in a hybrid system depends on the investment outlays and is the subject of ongoing optimization investigations. These studies will be supported by a model with a plug flow on the feed side and a countercurrent plug flow on the permeate side, presented in the current study and positively validated from experimental data.

## Figures and Tables

**Table 1:** Module permeance of carbon dioxide, nitrogen, and oxygen in UBE UMS-A5.

AQ, GPU m^2^	Feed Pressure, bar (a *)	288 K	293 K	298 K
CO_2_	1.8−7.5	19.6 ± 2.2	20.4 ± 2.0	21.7 ± 1.6
O_2_	2.0−7.5	3.5 ± 0.1	3.8 ± 0.1	4.5 ± 0.1
N_2_	3.0−7.5	0.39 ± 0.01	0.44 ± 0.02	0.48 ± 0.01

* An absolute pressure.

**Table 2:** Module permeance of carbon dioxide, nitrogen, and oxygen in Air Products PRISM PA1020.

AQ, GPU m^2^	Feed Pressure, bar (a *)	288 K	293 K	298 K
CO_2_	1.8−3.0	321.0 ± 3.9	342.2 ± 5.2	361.4 ± 6.1
O_2_	4.7−7.5	54.0 ± 1.0	61.6 ± 0.6	68.0 ± 1.1
N_2_	1.5−7.5	7.1 ± 0.3	8.4 ± 0.6	9.8 ± 0.5

* An absolute pressure.

**Table 3:** Module permeance of carbon dioxide, nitrogen, and oxygen in Air Products PRISM PA1020 and UBE UMS-A5 modules.

	Air Products, PRISM PA1020 AQ, GPU m^2^ (at 295 K)		UBE, UMS-A5 AQ, GPU m^2^ (at 296 K)	
	Year 0	Year 8	Year 0	Year 7
CO_2_	353	322	21.7	17.9
N_2_	9.33	8.97	0.457	0.404
O_2_	61.7	54.3	4.13	3.55

## Supplementary Materials

The following are available online at https://www.mdpi.com/2077-0375/10/11/309/s1, Table S1: Comparison of modules.
