# Supplementary material for: The Separative Performance of Modules with Polymeric Membranes for a Hybrid Adsorptive/Membrane Process of CO2 Capture from Flue Gas
Source: Membranes (Basel). 2020 Oct 28;10(11):309. doi: 10.3390/membranes10110309 (PMC7692737; doi:10.3390/membranes10110309)
Supplement: Supplementary file 1 [file membranes-10-00309-s001.pdf]

Electronic Supplementary Information

The separative performance of modules with polymeric membranes for a hybrid, adsorptive/membrane process of CO_2 capture from flue gas.

Aleksandra Janusz-Cygan [1,*], Jolanta Jaschik [1], Artur Wojdyła [1] and Marek Tańczyk [1]

Institute of Chemical Engineering, Polish Academy of Sciences, ul. Bałtycka 5, 44-100 Gliwice, Poland; jjaschik@iich.gliwice.pl (J.J.); artur.wojdyla@iich.gliwice.pl (A.W.); mtanczyk@iich.gliwice.pl (M.T.)
Correspondence: ajcygan@iich.gliwice.pl

Table S1. Comparison of modules

**Module type**	**PRISM PA1020**	**UMS-A5**
**Manufacturer**	Air Products	UBE
**Housing material**	ABS + aluminium	stainless steel
**Membrane material**	modified polysulfone	modified polyimide
**Length, mm**	660	680*
**Diameter of the wider part of the module / diameter of the working part**	53 / 42	47 / 29*
**Weight, kg**	0.7	2.2*
**Maximum inlet temperature, °C**	55	40
**Maximum inlet pressure, bar(g)**	15	9.9
**Maximum particle size in the feed gas, μm**	0.01	0.01
**Maximum oil content in the feed gas, ppm**	0.001	0.001
**Lifetime**	8-12	no data available

*) self-measurement
